# Promoting Latinx health equity through community-engaged policy and practice reforms in North Carolina

**DOI:** 10.3389/fpubh.2023.1227853

**Published:** 2023-11-23

**Authors:** Andrea Thoumi, Gabriela Plasencia, Farrah Madanay, Ethan Shih-An Ho, Caroline Palmer, Kamaria Kaalund, Nikhil Chaudhry, Amy Labrador, Kristen Rigsby, Adaobi Onunkwo, Ivan Almonte, Rosa Gonzalez-Guarda, Viviana Martinez-Bianchi, Rushina Cholera

**Affiliations:** ^1^Margolis Center for Health Policy, Duke University, Washington, NC, United States; ^2^Department of Family Medicine and Community Health, School of Medicine, Duke University, Durham, NC, United States; ^3^LATIN-19, Durham, NC, United States; ^4^Margolis Center for Health Policy, Duke University, Durham, NC, United States; ^5^Sanford School of Public Policy, Duke University, Durham, NC, United States; ^6^Pratt School of Engineering, Duke University, Durham, NC, United States; ^7^Trinity College of Arts & Sciences, Duke University, Durham, NC, United States; ^8^Fuqua School of Business, Duke University, Durham, NC, United States; ^9^School of Nursing, Duke University, Durham, NC, United States; ^10^Department of Pediatrics, School of Medicine, Duke University, Durham, NC, United States

**Keywords:** health equity, Latino/Hispanic people, COVID-19, community-engaged research, community-academic partnership

## Abstract

**Introduction:**

The Latinx Advocacy Team & Interdisciplinary Network for COVID-19 (LATIN-19) is a unique multi-sector coalition formed early in the COVID-19 pandemic to address the multi-level health inequities faced by Latinx communities in North Carolina.

**Methods:**

We utilized the National Institute on Minority Health and Health Disparities (NIMHD) Research Framework to conduct a directed content analysis of 58 LATIN-19 meeting minutes from April 2020 through October 2021. Application of the NIMHD Research Framework facilitated a comprehensive assessment of complex and multidimensional barriers and interventions contributing to Latinx health while centering on community voices and perspectives.

**Results:**

Community interventions focused on reducing language barriers and increasing community-level access to social supports while policy interventions focused on increasing services to slow the spread of COVID-19.

**Discussion:**

Our study adds to the literature by identifying community-based strategies to ensure the power of communities is accounted for in policy reforms that affect Latinx health outcomes across the U.S. Multisector coalitions, such as LATIN-19, can enable the improved understanding of underlying barriers and embed community priorities into policy solutions to address health inequities.

## Introduction

Growing evidence during the COVID-19 pandemic reinforced the association between disparate health outcomes and structural racism among historically marginalized populations (1–4). Populations who identify as Hispanic, Latino, or Latinx (herein Latinx) experienced disproportionate risk of COVID-19 disease burden, hospitalizations, and mortality compared to non-Hispanic, White populations (3, 5, 6). In addition, Latinx communities experienced significant declines in life expectancy (7). In North Carolina, Latinx communities represented over half of early COVID-19 cases despite comprising 10 percent of the state's total population (8).

Health inequities heightened during the pandemic reflect long-standing systemic exclusion from resources and opportunities to promote health (9–13). Systemic exclusion occurs when communities experience continuous and compounded legal, regulatory, and political injustices that hinder a population's ability to achieve health and wellbeing. For example, Latinx community members disproportionately lack health insurance coverage and live in neighborhoods with limited or unreliable access to a pharmacy, public transportation, or broadband Internet (4, 9, 13, 14). In addition, acculturative and socioeconomic stressors that stem from discriminatory immigratory practices, lack of stable employment, and racism contribute to declines in physical and mental wellbeing among Latinx populations (15). In North Carolina, Latinx essential workers are disproportionately represented in meat packing and food processing industries, which have limited workplace protections or paid sick leave. During the pandemic, these factors contributed to greater risk of exposure and inability to take time off when workers were sick. In addition, anti-immigrant sentiment, unpredictable immigration policy, and experiences related to family separation prevented Latinx individuals from seeking COVID-19 testing, vaccination, and treatment, or other public health benefits (16–19). For example, initial delivery of COVID-19 testing required government-issued identification or social security numbers, making resources inaccessible to Latinx community members without legal status (20).

Responding to local pandemic-related health inequities, Latina clinicians in Durham, North Carolina created the Latinx Advocacy Team and Interdisciplinary Network for COVID-19 (LATIN-19), a coalition of community, academic, healthcare, and policy stakeholders, that has met weekly since March 2020 and continues implementing its mission today (20, 21). Specifically, LATIN-19 was formed to address the multi-level health inequities faced by Latinx communities in North Carolina during the COVID-19 pandemic (22). These health inequities spanned disparities in COVID-19 mortality, morbidity, and risk of exposure, as well as inequitable access to COVID-19 testing, vaccination, therapeutics and other resources. Through weekly virtual meetings conducted by bilingual and bicultural coalition leaders, LATIN-19 has lifted community voices and created a bi-directional platform to increase community-engaged strategies in policy solutions (21, 22).

Community-academic coalitions, or partnerships among community-based participants including individuals or organizations with academic institutions, serve crucial roles to bring together community members, policymakers, and researchers across sectors to advance health equity in policy responses (23). Preliminary analysis suggests that community and policy interventions informed by LATIN-19 contributed to reducing the COVID-19 vaccine equity gap among Latinx populations in the state (21). North Carolina's COVID-19 vaccination strategies prioritized health equity through community partnerships, data, communication, and payment models, which mitigated the vaccination equity gap during the rollout of the vaccine (24, 25). By October 2021, Latinx populations in North Carolina were the ethnic group most likely to have been vaccinated with at least one dose (8). Despite the state's progress in improving health equity during its COVID-19 vaccination campaign, disparities in rates of COVID-19 therapeutic provision and booster vaccinations emerged in 2022. Latinx children were less likely to be vaccinated and Latinx community members were less likely to receive a booster or monoclonal antibodies (8). These continued disparities due to social and political determinants of health underscore the need for ongoing, long-term efforts to overcome established systemic barriers.

In this study, we examine barriers to COVID-19 testing and vaccination as identified by LATIN-19 meeting participants, assess the alignment of community and policy interventions to observed barriers, and recommend policy strategies to overcome identified challenges. We apply the National Institute on Minority Health and Health Disparities (NIMHD) Research Framework, an accepted tool for organizing complex factors associated with minority health and mapping gaps and opportunities that can inform systems-wide recommendations (26). The NIMHD Framework is an ecological model that captures different levels of influences on health disparities (individual, interpersonal, community, and societal) according to domains of influence (biological, behavioral, built and sociocultural environment, and health care system) and across the life course. Our study adds to the literature by identifying community-based strategies to ensure the power of communities is accounted for in policy reforms that address underlying structural inequities that affect the health of Latinx communities across the U.S.

## Materials and methods

### Data sources and assessment tool

We adopted the NIMHD Research Framework to systematically analyze 58 LATIN-19 meeting minutes from April 2020 through October 2021. These hour-long weekly meetings anchored LATIN-19′s response by providing a regular safe space for community members to voice concerns during the pandemic and for leaders from diverse sectors (e.g., medical, public health, social) to use these voices to inform change (22). Meetings were open to all participants, which included people with lived experiences. LATIN-19 meeting minutes also offered a longitudinal archive of robust, community-engaged discussions that occurred at different stages of the pandemic (e.g., masking guidance, testing access, vaccination rollout, back to school, etc.) allowing for in-depth consideration of evolving needs. Meeting minutes included input from LATIN-19 participants representing their own lived experiences or in their professional capacity in academic institutions, health care systems, public health departments, public school systems, CBOs, government, and faith communities.

Meeting minutes were taken initially by a trained and supervised medical student volunteer and later by a trained and supervised program coordinator. Meeting minutes were reviewed for accuracy by the meeting host. Therefore, all notes were taken by two individuals, lending relative consistency in style and selection of important ideas or discussions to include in the notes. Notes were stored in Box, a secure online service provided by the university. Meeting minutes offer a useful alternative in community settings with limited resources to record, transcribe, and translate weekly community-based meetings. We also utilized Press Releases released by the North Carolina Department of Health and Human Services (NCDHHS) as a data source.

### Study design and analysis

We conducted a directed content analysis by applying the NIMHD Framework's levels and domains of influence as our codebook (27). Using this framework-based deductive approach, nine team members coded data based on the NIMHD domains of influence in the first round of coding and the NIMHD levels of influence in the second round of coding (28). For each round, two coders independently coded each meeting minute and a third coder reviewed coding of all meeting minutes. Coding was performed in Microsoft Excel, instead of other commonly used Qualitative Data Analysis (QDA) tools, due to the number of coders, number of meeting minutes to review, and difficulties associated with blind coding across reviewers with typical QDA tools (29). The coding team met weekly to discuss coding discrepancies and build consensus on the application of the codebook to the meeting minutes.

Community interventions were defined as interventions led by community-based organizations (CBOs), whereas policy interventions were specific public health or health policy measures implemented by NCDHHS. Interventions that directly addressed an observed barrier identified in the LATIN-19 meeting minutes were considered an aligned intervention. Two team members reviewed Press Releases released by the NCDHHS between April 2020 and October 2021 to identify additional policy interventions that addressed community-identified barriers. Research was approved by the Institutional Review Board.

## Results

**Figure 1:**
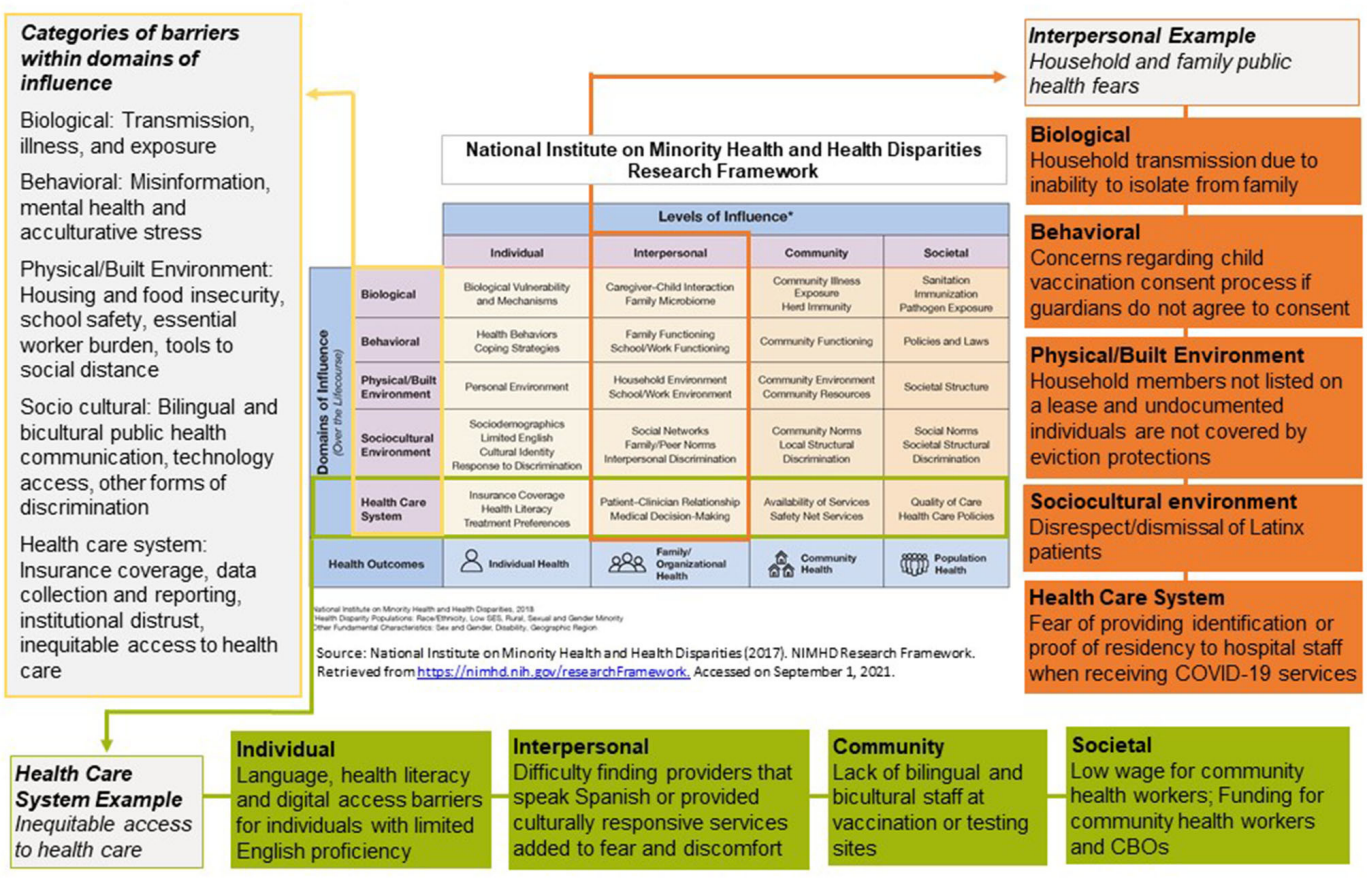
Illustrative examples of barriers identified in LATIN-19 meeting minutes using of the NIMHD research framework, April 2020–October 2021.

Our analysis highlighted several long-standing systemic barriers in health care access, social and political determinants of health, and discrimination that re-emerged and persisted during the COVID-19 pandemic. We also identified key community and policy interventions that addressed these barriers. Below, we discuss these barriers according to the NIMHD levels of influence: individual, interpersonal, community, and societal. The same barrier could manifest across different levels of influence, which are not mutually exclusive. Figure 1 illustrates how we applied the NIMHD Research Framework. The figure also demonstrates how barriers were further categorized within domains of influence: biological, behavioral, physical and built environment, sociocultural environment, and health care system. Table 1 summarizes barriers by level and domain of influence.

**Table 1:** COVID-19 barriers identified in LATIN-19 meeting minutes by levels and domains of influence (NIMHD Research Framework), April 2020–October 2021.

			**NIMHD research framework levels of influence**			
		**Categories of barriers within domains of influence**	**Individual**	**Interpersonal**	**Community**	**Society**
**NIMHD research framework domains of influence**	**Biological**		**Biological vulnerability and mechanisms**	**Caregiver-child interactions and family microbiome**	**Community illness, exposure, herd immunity**	**Sanitation, immunization, pathogen exposure**
		**Transmission, illness, and exposure**	**Masking fatigue and inconsistent use of masking**	**Household transmission due to inability to isolate from household members**	**High hospitalization rates in Latinx community early in pandemic; concerns about spread at local schools**	**Increased exposure risk due to working essential jobs and needing to continue to work for a steady income**
	**Behavioral**		**Health behaviors, coping strategies**	**Family functioning, school/work functioning**	**Community functioning**	**Policies and laws**
		**Misinformation**	**Vaccine deliberation due to misinformation about individual immunity following infection and safety of vaccines for children and pregnant women**	**Concerns regarding child vaccination consent process if guardians do not agree to consent**	**Vaccine deliberation due to misinformation about side effects from the vaccine and speed of development and approval process**	**Limited public health communication in accessible formats (e.g., at health literacy level, digital and paper, multilingual)**
		**Mental health and acculturative stress**	**Concerns about self-isolation, hospitalization, and separation from family in the event of a COVID-19 positive case (re-traumatizing event for many families)**	**Fear that accessing COVID-19 services would affect household immigration status Fear of family separation due to restrictive hospital visitor policies**	**Fear of seeking medical care due to Public Charge**	**Fear of deportation resulting from contact tracing and data collection**
	**Physical and built environment**		**Personal environment**	**Household environment, school/work environment**	**Community environment, community resources**	**Societal structure**
		**Housing insecurity**	**Housing instability as a contributor to negative health outcomes**	**Household members not listed on a lease and undocumented individuals are not covered by eviction protections**	**Difficulty finding appropriate housing to quarantine**	**Exclusion from state-provided housing programs due to immigration status**
		**Food insecurity**	**None observed**	**Concerns about household and childhood food insecurity**	**Lack of asset mapping of food resources**	**None observed**
		**School safety**	**None observed**	**Inability to maintain COVID-19 precautions in schools (e.g., limited facilities for outdoor eating, staff shortages, insufficient PPE supplies, space/staff for social distancing)**	**Child/nurse ratios at schools too high for testing**	**Not all Durham Public Schools had funding to implement universal masking**
		**Essential worker burden**	**Financial and employment-related burden due to essential jobs and confusion regarding employment protections for essential workers**	**Unable to miss work due to financial instability or work from home**	**Higher levels of uninsurance rates among essential workers**	**Lack of paid sick leave and PPE (e.g., masks)**
		**Tools to social distance**	**None observed**	**Difficult to social distance at home, at work, or in employer-provided housing (for farmworkers)**	**Inaccessible quarantine protocols to people with limited English proficiency**	**Inaccessible resources to be able to quarantine and isolate**
**NIMHD research Framework Domains of Influence**	**Sociocultural environment**		**Sociodemographics, limited english, cultural identity, response to discrimination**	**Social networks, family/peer norms, interpersonal discrimination**	**Community norms, local structural discrimination**	**Social norms, societal structural discrimination**
		**Bilingual and bicultural public health communication**	Scarce multilingual and accessible information to meet health literacy level for people with limited English proficiency	None observed	Culturally inaccurate and incorrectly translated resources especially if translated by machine-learning applications	NCCARE360 and MyChart initially not in languages other than English Community health worker (CHW) training only offered in English
		**Technology access**	Confusion regarding vaccine eligibility among individuals without digital access	Difficulty accessing telehealth resources due to lack of WiFi among Latinx farmers	None observed	Digital literacy and digital divide preventing access to telehealth services or online resources
		**Other forms of discrimination**	Limited transportation options to access events	Disrespect or dismissal of Latinx patients	Lack of resources and limited knowledge about what would happen to asylum seekers complicated seeking testing services Lack of support provided to patients with limited English proficiency after hospital discharge on weekends	Difficulty applying to long-term acute care hospitals (LTACHs) for people lacking legal status or mixed-status households Trauma and systemic exclusion from health systems Fear of being monitored or tracked due to immigration status
**NIMHD research framework domains of influence**	**Health care system**		**Insurance coverage, health literacy, treatment preferences**	**Patient-clinician relationship, medical decision making**	**Availability of services, safety net services**	**Quality of care, health care policies**
		**Insurance coverage**	Reluctance to seek treatment due to unknown costs	None observed	High copays for COVID-19 testing and medical care	Medicaid churn due to fears of public charge and family separation, recertification requirements, and NC Medicaid change to Medicaid managed care
		**Data collection and reporting**	None observed	Fear of providing identification or proof of residency to hospital staff when accessing COVID-19 medical services	Lack of race/ethnicity data created concern that reported cases were inaccurate	Delayed contact tracing and testing result dissemination Lack of data transparency regarding use of data (e.g., concerns about data sharing with U.S. Immigration and Customs Enforcement)
		**Institutional distrust**	Requirement of proof of identification initially required at many testing sites	Distrust in the medical system	Community members asked to participate in clinical trials, yet do not have equitable access to health care	Historical and contemporary distrust of health care system and data tracking
		**Inequitable access to health care**	Language, health literacy, and digital access barriers for individuals with limited English proficiency	Difficulty finding providers that spoke Spanish or provided culturally responsive services added to fear and discomfort	Lack of bilingual and bicultural staff at vaccination or testing sites	Low wage for CHWs Limited funding for CHWs and community-based organizations (CBOs)
**Health outcomes**			**Individual health**	**Family/organizational health**	**Community health**	**Population health**
			Disproportionately high COVID-19 exposure, cases, and mortality racial and ethnic disparities in testing and vaccination rates			

Source: Authors' analysis.

### Participation represented in meeting minutes

Average LATIN-19 meeting participation during the study period was 76 participants per meeting (range 40–100). Initial average participation was 40 people in May 2020 and quickly increased to 85 people by July 2020. With the exception of October 2020, August 2021, and October 2021, all other months in the study period exhibited a monthly average participation rate above 70 people, an indicator of consistent and active participation. A peak monthly average occurred in February 2021–April 2021 (80–95 monthly averages), which was the time period of initial COVID-19 vaccinations. Broadly, LATIN-19 meetings included discussions of barriers Latinx community members experienced in North Carolina, as well as solutions identified by participants. LATIN-19 does not collect descriptive data on individual participants.

### Individual barriers: lack of transportation and technology, limited language concordance, employment uncertainty, and financial consequences

Transportation and out-of-pocket cost barriers made accessing COVID-19 testing and vaccinations challenging. Individuals were unsure if transportation to testing sites would be covered, were worried about the cost of receiving testing or vaccinations without health insurance, and did not trust that vaccinations were completely free given confusing messaging. Furthermore, individuals feared unknown financial consequences due to limited paid time off and lack of clarity regarding who qualified as an essential worker. Confusion repeatedly peaked following intermittent changes to quarantine, mask, and social distancing requirements. Stress related to self-isolation, hospitalization, and separation from family due to quarantine protocols impacted mental health. In addition, individuals reported difficulties finding updated COVID-19 guidelines or information without a smartphone or Internet access. Another concern was the lack of high-quality, linguistically accessible, and culturally relevant COVID-19 information available for people whose preferred language was not English.

### Interpersonal barriers: inability to quarantine at-home, miss work, and social distance at work or school

Precarious food, housing, and economic realities made mitigating COVID-19 transmission in households difficult as working-age individuals were unable to miss work or work from home. Further, as multi-generational and multi-family households are common in many Latinx communities, some families reported household transmission due to lack of space and inability to quarantine within their home. Cultural norms to care for older adult family members at home also placed households at higher risk of transmission. As schools transitioned back to in-person classes, most local public schools did not have facilities for outdoor eating, and faced staffing shortages, which reduced the ability to enforce recommended COVID-19 precautions. Concerns about potential eviction compounded household stress related to employment and transmission, especially if household members lacked legal status or were not listed on the lease. Federal eviction protections did not apply to these household members further worsening housing insecurity.

### Community barriers: vaccine deliberation, concerns about public charge, limited bilingual or bicultural information and providers

Historical and contemporary mistrust of health systems and government institutions coupled with limited availability of information in Spanish contributed to vaccine deliberation, or considering information before making a decision, at the community level (30). Modifications to North Carolina Medicaid coverage during the pandemic, including the transition to Medicaid managed care in July 2021 and changes in medical billing codes, increased concerns about disruptions in Medicaid coverage and out-of-pocket costs for COVID-19 testing, vaccination, and hospital treatment. Importantly, changes to the Public Charge Inadmissibility Rule, which refers to receipt of public benefits that can negatively impact eligibility for lawful permanent residence, caused fear and concern regarding whether use of Medicaid benefits or COVID-19 treatment would impact immigration status. Ubiquitous language barriers further compounded existing mental health and acculturative stressors. Few sources of bilingual information existed for community members. Even when available, these resources were not accessible to community members with limited access to technology or limited health literacy; thus, community members increasingly relied on informal and often erroneous sources of information. Other barriers spanned social determinants of health, including food insecurity, and forms of discrimination. For example, families were referred to social service organizations that often lacked culturally trained staff or had limited availability of Spanish-speaking providers.

### Societal barriers: data collection and reporting, fears of data use due to deportation risk, inaccessible services for underinsured or uninsured populations

Initially, data for COVID-19 testing was not publicly available by race and ethnicity, which hindered the development of interventions to overcome specific barriers that Latinx community members experience. Lack of clarity regarding data privacy and data sharing among state and federal agencies resulted in delayed or avoided treatment. Community members expressed fear of deportation due to the possibility of U.S. Immigration and Customs Enforcement using personal identification or proof of residency information collected at testing and vaccination sites in immigration status cases. These fears contributed to ongoing stressors related to immigration status and surveillance, resulting in reinforced trauma and reduced access to COVID-19 resources. Lastly, lack of insurance and long wait times (2–3 months) to receive Medicaid coverage led to inaccessible COVID-19-related services and increased stress from unknown out-of-pocket costs.

### Alignment of community and policy interventions to observed community-identified barriers

Most observed barriers were addressed through at least one type of intervention (e.g., community or policy). Some categories within domains of influence, such as transmission, illness, and exposure; tools to social distance; bilingual and bicultural health communication; and equitable access to health care, were addressed by both community and policy interventions. However, some observed barriers lacked either a policy or community intervention, or both. For example, the following categories within domains of influence included at least one level of influence lacking an intervention: mental health and acculturative stress, essential worker burden, technology access, discrimination, insurance coverage, data collection and reporting, and institutional distrust. Specifically, neither community nor policy interventions addressed any of the observed barriers related to essential worker burden, such as financial burden, inability to miss work, and lack of paid sick leave. Several discrimination barriers also remained unaddressed by either community or policy interventions, such as disrespect of Latinx patients, lack of support for patients whose preferred language was not English after hospital discharge, trauma, and systemic exclusion from health systems.

**Figure 2:**
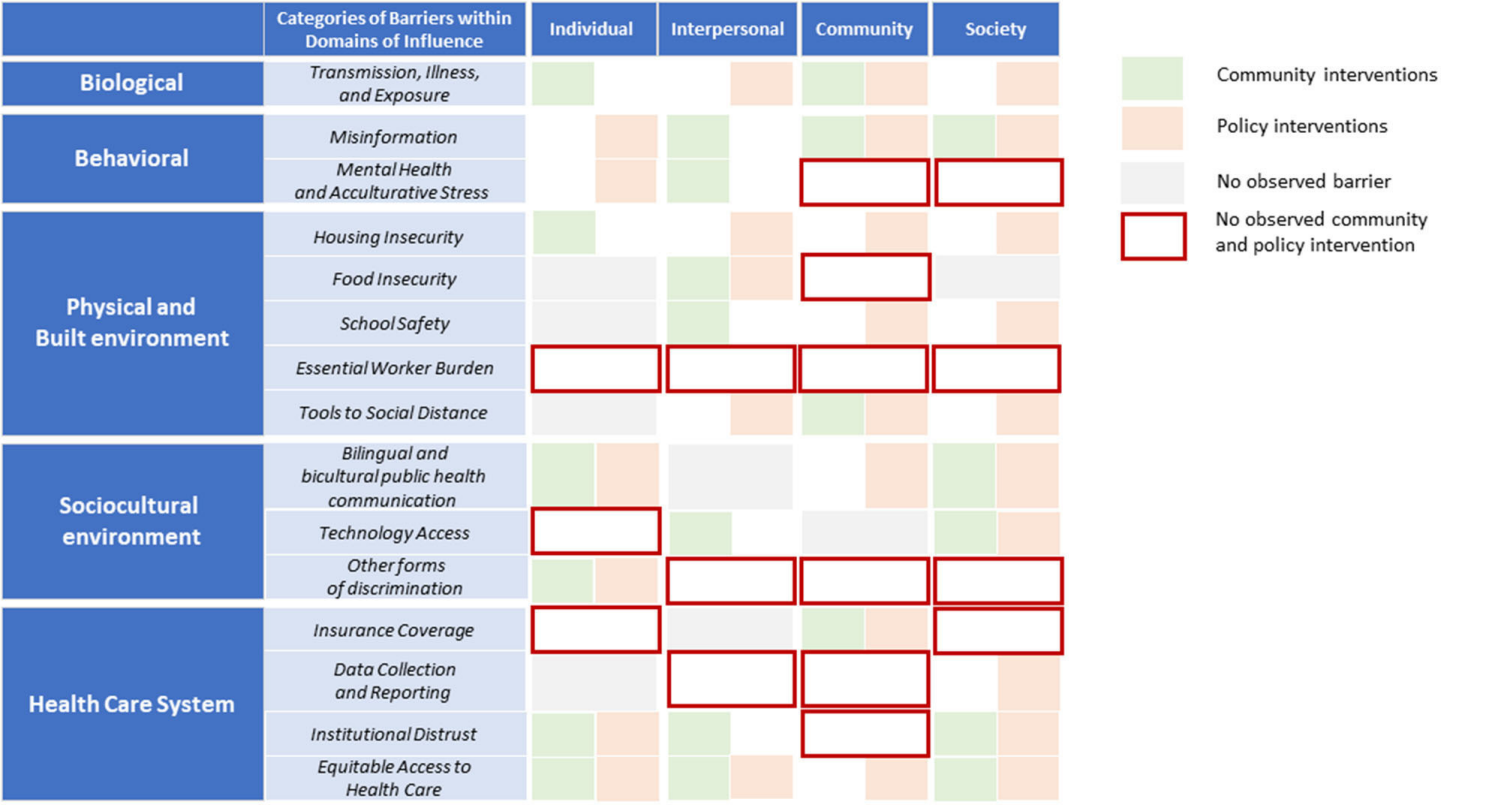
COVID-19 community and policy interventions by levels and domains of influence (NIMHD research framework), April 2020–October 2021. Policy interventions that did not directly address observed barriers noted in the LATIN-19 meeting minutes were not included in this analysis. Source: Authors' analysis.

Community interventions leveraged expertise through iterative and bi-directional decision-making processes. These solutions focused on connecting Latinx community members to the right COVID-19 information, resources, and support systems while overcoming systemic barriers. Policy interventions focused primarily on amending and expanding existing health care infrastructure to improve access to services. These solutions leveraged policy change to better integrate social and health services and remove restrictions to access to existing public health services. In several instances, policy and community interventions jointly addressed the same observed barriers. For example, both community and policy interventions were developed to address challenges related to COVID-19 vaccine misinformation. Community-based organizations increased Spanish-speaking staff at community outreach events and developed bilingual websites on COVID-19 resources, whereas NCDHHS developed Spanish community health worker trainings and Spanish information campaigns. Figure 2 illustrates community and policy interventions organized by the barriers they address (see Supplementary material for a detailed summary of solutions).

## Discussion

Our findings advance the literature on community-engaged strategies to promote health equity among Latinx populations experiencing systemic exclusion from health care, public health, and social services. As prior research shows, interventions to improve health equity require coordination and partnerships across sectors, authentic community engagement, and removal of policies that lead to inequitable health outcomes (21, 31). Here, we analyzed discussions from LATIN-19, a community-academic coalition that has regularly convened community members, researchers, public health leaders, and policymakers to engage in meaningful communication and mutual goal-setting since March 2020. Our application of the NIMHD Research Framework facilitated a comprehensive assessment of complex and multidimensional factors contributing to health disparities in Latinx health while centering on community voices and perspectives. To our knowledge, we are the first research team to use this framework as a tool to assess community-identified pandemic-related barriers and policy implications among Latinx communities (32, 33). We found that barriers clustered at the community and society levels, and community and policy interventions piloted during the COVID-19 pandemic focused on increasing access to social supports and developing and disseminating information in Spanish.

Importantly, most barriers identified in our study are not new or unique to the pandemic, underscoring that extensive documentation of systemic inequities is insufficient to lead to policy action. For example, access to linguistically appropriate resources has been a longstanding challenge, and worsened with the rapidly changing recommendations and guidelines during the pandemic (9–11). Similarly, pre-existing gaps in access to mental health care for Latinx populations widened during the pandemic (34, 35). Meanwhile, pandemic-related stressors disproportionately impacted the mental health of historically marginalized populations. For example, new modalities of family separation emerged during the pandemic. Families with household members without legal status were fearful of seeking medical care due to lack of communication once a family member was admitted to a hospital, restrictive visitation policies, and language barriers. Additional concerns surrounded logistics of obtaining remains of loved ones for transportation back to the country of origin. LATIN-19 participants noted that health systems often lack bilingual and culturally sensitive staff. Policy changes that resulted in more frequent communication to families when loved ones were hospitalized helped assuage fears of a “desaparecido,” or disappeared individual, which is a common familial trauma among many Latin Americans across the region.

A near-term policy implication to reduce discrimination and institutional distrust is to expand the bilingual and bicultural workforce by including community health workers and CBOs as partners in public health or health care transformation efforts that uphold cultural humility. Additional policies outside of the health care domain, such as those removing requirements for presenting government identification or proof of residency when seeking COVID-19 testing or vaccinations, directly addressed community fears of tracking, separation, deportation, and discrimination. Such measures were disseminated by trusted community organizations and led to improved vaccination coverage and, if implemented earlier, could have potentially prevented hospitalizations and deaths from COVID-19. These solutions were developed when community members and policy makers engaged in longitudinal, bi-directional information sharing during the pandemic. In response to the end of the Public Health Emergency, such solutions can be adapted to continue to remove systemic access barriers, address social determinants of health, and support community-based care delivery models as a fundamental prevention strategy for public health.

Second, alignment of policy and community interventions to address different components of the same barriers in tandem can lead to more comprehensive change. Community interventions tended to focus on reducing language barriers and increasing community-level access to social supports while policy interventions focused on creating the infrastructure to increase services to slow the spread of COVID-19. Community-based organizations and community health workers led efforts to distribute food, increase transportation, and increase access to community-based COVID-19 testing and vaccinations. In addition, community interventions advanced bilingual messaging to clarify that all individuals, regardless of immigration status, could access COVID-19 testing and vaccination. Simultaneously, policy interventions included increasing funding for CBOs to conduct COVID-19 testing and vaccinations, as well as advancing state-wide programs (e.g., North Carolina Integrated Care for Kids and NCCARE360) to increase access to social supports.

Our findings highlight the critical role that community-based partners play in building trust, expanding access to services, and communicating culturally relevant information. Community-academic coalitions, such as LATIN-19, can also promote policy or programmatic changes, such as advocating for the implementation of MyChart, an electronic medical record system, in Spanish to make it easier for patients to track and view health information. Formalizing such coalitions as key partners to policymakers can ensure that interventions, regardless of type, do not have unintended consequences or inflict harm on community members. Policy implications include closing funding gaps based on periodic analysis of locally available community-level data to facilitate community-engaged research. These data can inform real-time priority setting and resource allocation to interventions that are aligned to community priorities and lead to more sustainable and responsive reforms. Funding could support transcription and translation of community discussions into quantifiable data, training of community health workers to increase data collection capacity, and community advisory boards to inform which health system or public health performance measures are aligned to community priorities.

Third, addressing systemic barriers rooted in structural racism requires multifaceted, culturally congruent solutions across multiple levels and domains of influence. While many observed barriers included common health policy or social determinant of health domains (e.g., insurance coverage, food, employment, health literacy, access to services, medical distrust), other barriers included fears related to immigration status, discrimination, and acculturative stress. Longer-term policy implications include addressing the social and structural determinants of health that lead to health disparities in Latinx communities, such as lower access to employment and health benefits. This will require policy action, such as expanding insurance coverage, but also extending to policies that reduce acculturative stress. For example, community-based outreach is needed to reverse the chilling effect of misinformation related to Public Charge, which has deterred families from seeking medical services or enrolling in public benefits for which family members may be eligible (e.g., Medicaid, SNAP, or WIC). Continued monitoring and sharing of health data by race and ethnicity and by public benefit program would help identify gaps that require community-based partnerships. Further, the pandemic has stressed how health systems and policy makers need to financially support community partners that elevate community priorities to the level of large health systems, private sector leaders, and health departments. Policies that redirect financial resources to community partners could be achieved by sharing savings from alternative payment models that incorporate incentives for equitable outcomes.

Although not the focus of our study, future efforts could explore which types of interventions are most effective in addressing community priorities to improve public health. In our study, we considered interventions aligned if they addressed an observed barrier; however, we were not able to determine if a barrier was best addressed through a policy intervention, a community intervention, or both. Efficiencies could occur from policy and community interventions addressing the same domain of influence but different levels of influence. For example, a CBO is likely to have the local knowledge and cultural humility to address individual- or household-level concerns related to mental health and acculturative stress, but a policy measure to address these areas could include expansion of behavioral telehealth services. Efficiencies could also occur by a policy intervention collaborating with community-based partners to address a single barrier. For example, increasing culturally informed information could include contracting CBOs to create bilingual handouts for public health guidance.

Strong multisector coalitions, such as LATIN-19, can help bridge silos across academic institutions, CBOs, and policymakers, and lead to more effective collaboration across stakeholders. LATIN-19 continues to meet today to address well-established and persistent health inequities including improving health literacy, increasing participation of Latinx individuals in clinical trials, bolstering capacity for community health workers to engage in clinical research and care navigation, and increasing insurance enrollment among Latinx populations. Key lessons learned from LATIN-19 experience include actively and intentionally centering community perspectives in the design, implementation, and assessment of policy or community interventions; addressing operational silos that stem from different and multiple sectors (e.g., health, social, education, as well as profit or non-profit status) by encouraging continuous and ongoing discussion to solve problems and create solutions; and developing multi-sectoral interventions to adequately respond to underlying systemic barriers that lead to poor health outcomes.

This study has limitations. Meeting minutes did not include direct quotes from meeting participants. The presentation topics of the invited guest speakers may have also influenced the ensuing community-based discussions. Furthermore, meeting minutes excluded the perspectives of community members that could not attend weekly LATIN-19 meetings. Lastly, our analysis did not include an impact or effectiveness assessment of the interventions identified in the meeting minutes. Therefore, we are unable to draw causal inferences between identifying a specific issue by LATIN-19 and implementation of a policy or community solution or intervention.

## Conclusions

Multisector community-academic coalitions, such as LATIN-19, enable the improved understanding of underlying barriers, community priorities, and solutions to address inequities. Policies that center historically marginalized voices and build on the power of community-academic coalitions can results in structural changes that promote health equity during public health crises. Further, sustainable and comprehensive policy and community interventions are needed to continue addressing systemic barriers and pandemic-related stressors that have negatively affected Latinx communities. Lessons from North Carolina can inform the national health policy discourse on how to embed community-engaged approaches in health policies to reduce marginalization and systemic exclusion that result in inequitable health outcomes.

## Data availability statement

The original contributions presented in the study are included in the article/Supplementary material, further inquiries can be directed to the corresponding author.

## Author contributions

AT: conceptualization, methodology, formal analysis, funding acquisition, writing–original draft, and supervision. GP: conceptualization, methodology, formal analysis, funding acquisition, validation, and writing–original draft. FM: methodology, formal analysis, validation, data curation, project administration, and writing–original draft. EH, CP, and KK: formal analysis, writing–review and editing, and visualization. NC, AL, KR, and AO: formal analysis and writing–review and editing. IA and RC: conceptualization and writing–review and editing. RG-G: conceptualization, methodology, and writing–review and editing. VM-B: conceptualization, writing–review and editing, resources, and funding acquisition. All authors contributed to the article and approved the submitted version.
