# Supplementary material for: Promoting Latinx health equity through community-engaged policy and practice reforms in North Carolina
Source: Front Public Health. 2023 Nov 23;11:1227853. doi: 10.3389/fpubh.2023.1227853 (PMC10701733; doi:10.3389/fpubh.2023.1227853)
Supplement: Supplementary file 1 [file Data_Sheet_1.pdf]

Supplemental File: COVID-19 Community and Policy Solutions to Barriers Identified in LATIN-19 Meeting Minutes by Levels and Domains of Influence (NIMHD Research Framework), April 2020 – October 2021

			**NIMHD Research Framework Levels of Influence**			
		**Categories of Solutions within Domains of Influence**	**Individual**	**Interpersonal**	**Community**	**Society**
**NIMHD Research Framework Domains of Influence**	**Biological**		**Biological Vulnerability and Mechanisms**	**Caregiver-Child Interactions and Family Microbiome**	**Community Illness, Exposure, Herd Immunity**	**Sanitation, Immunization, Pathogen Exposure**
		Transmission, Illness, and Exposure	**C:** CBOs explain pandemic has not ended, stress importance of masks	**P:** RFA for COVID isolation housing program extended (1); NCDHHS creates online tools in English/Spanish to monitor symptoms (2); implements Isolation Support Program (3)	**C:** CBOs partner with schools and churches to increase access to vaccination; offer transportation; host mobile events<br><br>**P:** NCDHHS selects vendors to hire CHWs (4); RADx funding for community outreach (5)	**P:** State opens vaccine eligibility to frontline essential workers (Group 3) (6)
	**Behavioral**		**Health Behaviors, Coping Strategies**	**Family, School, Work Functioning**	**Community Functioning**	**Policies and Laws**
		Misinformation	**P:** NCDHHS creates English/Spanish website and phone line to help answer questions about COVID-19 vaccinations (1)	**C:** CBOs call parents and school employees to inquire about providing vaccines in schools	**C:** LATIN-19 publishes vaccine distribution data on websites<br><br>**P:** NCDHHS creates English/Spanish website and phone line to help answer questions about COVID-19 vaccinations (1), tests messaging with Latinx focus groups (2), uses social media influencers to counter misinformation (2)	**C:** CBOs add Spanish-speaking providers to media pieces, translate MyChart into Spanish, increase Spanish-speaking community outreach staff, develop Spanish/English bilingual website and social media<br><br>**P:** NCDHHS develops Spanish CHW trainings (3) and videos (4); State agencies receive grants to improve telehealth in Appalachian region (5); Gov. Cooper establishes Office of Digital Equity and Literacy (6)
		Mental Health and Acculturative Stress	**P:** NCDHHS publishes on COVID-19 and behavioral health, resources for behavioral health services, helplines, and webinar recordings (7)	**C:** CBOs ensure people understand access to vaccination does not impact immigration status	None identified	None identified
	**Physical and Built Environment**		**Personal Environment**	**Household, School, Work Environment**	**Community Environment, Community Resources**	**Societal Structure**
		Housing Insecurity	**C:** El Centro Hispano gives $155k to help with rent and utilities	**P:** Gov. Cooper passes executive order clarifying CDC eviction moratorium to prevent unwanted evictions (1)	**P:** NCDHHS announces a FEMA-approved non-congregate sheltering program (2)	**P:** Gov. Cooper passes executive order clarifying CDC eviction moratorium, including for undocumented people (1,3)
		Food Insecurity	None identified	**C:** CBOs La Semilla and El Centro Hispano distribute food	None identified	None identified

**NIMHD Research Framework Domains of Influence**				**P:** Healthy Opportunities Pilots serve food across pilot regions (4)		
		School Safety	None identified	**C:** Durham LEAP offers in-person and virtual tutoring for children; implements precautions inside preschools	**P:** DPS funds more school nurses (6)	**P:** NC school districts implement universal masking in schools and on buses (7)
		Essential Worker Burden	None identified	None identified	None identified	None identified
		Tools to Social Distance	None identified	**P:** NCDHHS implements North Carolina Farmworker Health Program to reduce transmission of COVID-19 in migrant farmworker camps (7)	**C:** CBOs improve multilingual quarantine resources, add Spanish-speaking providers to media pieces, translate MyChart into Spanish, increase Spanish-speaking outreach staff, develop Spanish/English website and social media platform<br><br>**P:** NCDHHS develops Spanish CHW trainings (8), creates Spanish/English online tools for COVID-19 testing and symptom monitoring (9	**P:** RFA for COVID isolation housing program extended (10); NCDHHS creates Spanish/English online tools for COVID-19 testing and symptom monitoring (9)
	**Sociocultural Environment**		**Sociodemographics, Limited English, Cultural Identity, Response to Discrimination**	**Social Networks, Family/Peer Norms, Interpersonal Discrimination**	**Community Norms, Local Structural Discrimination**	**Social Norms, Societal Structural Discrimination**
		Bilingual and Bicultural Public Health Communication	**C:** Increase Spanish-speaking outreach staff<br><br>**P:** NCDHHS develops Spanish website and tools for COVID-19 symptom monitoring, testing, and quarantining (1)	None identified	**P:** NCDHHS partners with over 36 organizations to ensure COVID-19 materials are translated accurately and culturally competently (2)	**C:** Duke translating MyChart to Spanish<br><br>**P:** NCDHHS develops Spanish CHW trainings (3)
		Technology Access	None identified	**C:** East Carolina receives grant to expand Internet connectivity for farmworkers and their families (4)	None identified	**C:** CBOs raise awareness and help people create MyChart accounts<br><br>**P:** NCCC System supports Digital Literacy Skills Project with NC 2020 COVID-19 Recovery Act and Title II funds (5)
		Other Forms of Discrimination	**C:** CBOs advertise transportation options<br><br>**P:** NCDOT and NCDHHS fund transit rides to vaccine sites (6); NCDHHS Support Services Program provides transportation to testing/medical	None identified	None identified	None identified

			visits after someone tests positive (7)			
NIMHD Research Framework Domains of Influence	Health Care System		**Insurance Coverage, Health Literacy, Treatment Preferences**	**Patient-Clinician Relationship, Medical Decision Making**	**Availability of Services, Safety Net Services**	**Quality of Care, Health Care Policies**
		Insurance Coverage	None identified	None identified	**C:** Education for providers on coding so patients not billed for COVID-19 tests/care<br><br>**P:** NC Medicaid offers optional COVID-19 testing program to cover costs of testing, treatment, and vaccines for eligible uninsured citizens (1)	None identified
		Data Collection and Reporting	None identified	None identified	None identified	**P:** NCCARE360 platform enables health organizations/CBOs to securely share client information and track outcomes together (3)
		Institutional Distrust	**C:** CBOs educate about right to deny ID requests for testing and vaccination; create "MyRights" card for Latinx people to show they do not need ID<br><br>**P:** NCDHHS launches Community Testing in High-Priority and Marginalized Populations Initiative (4); CDC affirms US citizenship not a condition for vaccination (5)	**C:** Hospitals partner with public schools and churches to improve vaccine distribution equity, bring in trusted community members to inform rural areas about COVID-19 (6)	None identified	**C:** CBOs develop a survey to capture feelings of distrust around contact tracing<br><br>**P:** NCCARE360 enables health organizations/CBOs to securely share client information and track outcomes together (3)
		Inequitable Access to Health Care	**C:** CBOs raise awareness and help people create MyChart accounts<br><br>**P:** NCCC System Office supports the Digital Literacy Skills Project with NC 2020 COVID-19 Recovery Act funds and Title II funds (7)	**C:** Increase Spanish-speaking and culturally familiar outreach staff, especially at community events; focus on communication equity, rather than interpreter/translation services<br><br>**P:** NCDHHS develops Spanish CHW training (8)	**P:** NCDHHS pushes to include bilingual staff or volunteers from partner organizations to help interpret for patients that do not speak English as a primary language (9)	**C:** Vaccine clinics hire more CHWs<br><br>**P:** RADx-UP post $50K funding opportunity for community testing/outreach/education (10); Private public partnership with NCDHHS increases funding for CBOs to increase vaccinations of BIPOC people (11)
**Health Outcomes**			**Individual Health**	**Family/Organizational Health**	**Community Health**	**Population Health**
			**Disproportionately high COVID-19 exposure, cases, and mortality**<br>**Racial and ethnic disparities in testing and vaccination rates**			

Notes: C = Community, P = Policy
Source: Authors’ analysis

References

Biological

1 https://files.nc.gov/ncdhhs/COVID%20Support%20Services%2007312020%20FINAL.pdf
2 https://www.ncdhhs.gov/news/press-releases/2020/07/16/governor-cooper-announces-ncdhhs-spanish-language-website-and-tools-check-covid-19-symptoms
3 https://www.ncdhhs.gov/news/press-releases/2020/08/25/ncdhhs-announces-covid-19-support-services-program-individuals-isolation-or-quarantine
4 https://www.ncdhhs.gov/news/press-releases/2020/08/07/ncdhhs-selects-vendors-supervise-250-new-community-health-workers-50-counties-covid-19-hot-spots
5 https://news.unchealthcare.org/2021/03/radx-up-covid-19-rapid-test-pilot-program-and-community-engagement-initiatives/
6 https://governor.nc.gov/news/press-releases/2021/03/02/gov-cooper-announces-frontline-essential-workers-eligible-vaccination-beginning-march-3

Behavioral

1 https://www.ncdhhs.gov/news/press-releases/2021/02/16/ncdhhs-adds-new-resources-and-tools-spanish-language-covid-19-vaccination-website
2 https://covid19.ncdhhs.gov/media/675/open
3 https://www.durhamnc.gov/4617/Durham-Vaccine-Equity-Program
4 https://covid19.ncdhhs.gov/media/1482/download
5 https://it.nc.gov/news/press-releases/2020/10/19/state-agencies-receive-grants-improve-telehealth-digital-inclusion-appalachian-region
6 https://it.nc.gov/news/press-releases/2021/07/01/governor-cooper-establishes-nations-first-office-digital-equity-and-literacy
7 https://www.ncdhhs.gov/divisions/mental-health-developmental-disabilities-and-substance-abuse/covid-19-and-behavioral-health#for-providers

Physical and Built Environment

1 https://governor.nc.gov/news/press-releases/2020/10/28/governor-cooper-signs-executive-order-strengthen-eviction-prevention-and-help-renters-stay-their
2 https://covid19.ncdhhs.gov/information/housing-and-sheltering/non-congregate-sheltering
3 https://sites.duke.edu/latinohealthroundtable/2020/11/04/hope-program-offers-rent-relief-payments-and-utility-assistance-across-north-carolina/
4 https://www.ncdhhs.gov/about/department-initiatives/healthy-opportunities/healthy-opportunities-pilots
5 https://www.ncsota.org/wp-content/uploads/2021/10/CDPHFAQsaboutCOVID-19andSchools.pdf
6 https://www.ednc.org/resource-tracking-nc-school-district-mask-mandates/
7 https://covid19.ncdhhs.gov/media/363/open
8 https://www.durhamnc.gov/4617/Durham-Vaccine-Equity-Program
9 https://www.ncdhhs.gov/news/press-releases/2020/07/16/governor-cooper-announces-ncdhhs-spanish-language-website-and-tools-check-covid-19-symptoms

10 https://files.nc.gov/ncdhhs/COVID%20Support%20Services%2007312020%20FINAL.pdf

Sociocultural Environment

1 https://www.ncdhhs.gov/news/press-releases/2020/07/16/governor-cooper-announces-ncdhhs-spanish-language-website-and-tools-check-covid-19-symptoms
2 https://www.ncdhhs.gov/news/press-releases/2021/04/20/ncdhhs-nc-counts-coalition-announce-500k-grants-support-equitable-covid-19-response
3 https://www.durhamnc.gov/4617/Durham-Vaccine-Equity-Program
4 https://www.farmworkerhealthliteracy.org/
5 https://www.nccommunitycolleges.edu/college-and-career-readiness/digital-literacy
6 https://www.ncdhhs.gov/news/press-releases/2021/01/21/ncdot-and-ncdhhs-announce-solution-help-fund-transit-rides-and-covid-19-vaccine-sites
7 https://www.ncdhhs.gov/news/press-releases/2020/08/25/ncdhhs-announces-covid-19-support-services-program-individuals-isolation-or-quarantine

Health Care System

1 https://medicaid.ncdhhs.gov/about-us/covid-19-guidance-and-resources/providers/covid-19-guidance-resources-medicaid-beneficiaries
2 https://ncchildcare.ncdhhs.gov/Portals/0/documents/pdf/C/COVID-19_Child_Care_Payment_Policies.pdf?ver=f0R-HXq4bGwjaE7aHWjF3Q%3d%3d
3 https://governor.nc.gov/news/north-carolina-creates-nation%E2%80%99s-first-statewide-infrastructure-connecting-healthcare-and-human
4 https://www.ncdhhs.gov/news/press-releases/2020/07/07/ncdhhs-deploy-300-free-testing-sites-underserved-communities
5 https://www.cdc.gov/vaccines/covid-19/citizenship-residency-position.html
6 https://www.northcarolinahealthnews.org/2022/08/04/trusted-community-messengers-data-key-in-north-carolinas-journey-to-vaccine-equity/
7 https://www.nccommunitycolleges.edu/college-and-career-readiness/digital-literacy
8 https://www.durhamnc.gov/4617/Durham-Vaccine-Equity-Program
9 https://covid19.ncdhhs.gov/media/2491/open
10 https://news.unchealthcare.org/2021/03/radx-up-covid-19-rapid-test-pilot-program-and-community-engagement-initiatives/
11 https://www.ncdhhs.gov/news/press-releases/2021/09/16/healthier-together-announces-additional-500k-grants-support-equitable-covid-19-response-north
